# UV-Accelerated Synthesis of Gold Nanoparticle–Pluronic Nanocomposites for X-ray Computed Tomography Contrast Enhancement

**DOI:** 10.3390/polym15092163

**Published:** 2023-05-01

**Authors:** Deizilene S. B. Gomes, Leonardo G. Paterno, Aline B. S. Santos, Debora P. P. Barbosa, Beatriz M. Holtz, Maysa R. Souza, Rafaianne Q. Moraes-Souza, Aisel V. Garay, Laise R. de Andrade, Patricia P. C. Sartoratto, Damien Mertz, Gustavo T. Volpato, Sonia M. Freitas, Maria A. G. Soler

**Affiliations:** 1 Universidade de Brasilia, Instituto de Física, Laboratório de Nanofilmes e Nano Dispositivos, Brasilia-DF 70910-900, Brazil; 2 Instituto Federal de Educação, Ciencia e Tecnologia de Rondonia, Ji-Parana-RO 76900-730, Brazil; 3 Universidade de Brasilia, Instituto de Quimica, Laboratorio de Pesquisa em Polimeros e Nanomateriais, Brasilia-DF 70910-900, Brazil; 4 Federal University of Mato Grosso, Institute of Biological and Health Sciences, Laboratory of System Physiology and Reproductive Toxicology, Barra do Garças-MT 78605-091, Brazil; 5 Universidade de Brasilia, Instituto de Ciências Biológicas, Departamento de Biologia Celular, Laboratório de Biofisica Molecular, Brasilia-DF 70910-900, Brazil; 6 Universidade de Brasilia, Instituto de Ciências Biologicas, Brasilia-DF 70910-900, Brazil; 7 Universidade Federal de Goias, Instituto de Quimica, Goiania-GO 74690-900, Brazil; 8 Institut de Physique et Chimie des Materiaux de Strasbourg (IPCMS), UMR-7504 CNRS-Universite de Strasbourg, 23 rue du Loess, BP 34, CEDEX 02, 67034 Strasbourg, France

**Keywords:** gold nanoparticle, Pluronic F127, nanocomposites, UV accelerated synthesis, X-ray computed tomography, cytotoxicity, maternal and fetal toxicity assays

## Abstract

Eco-friendly chemical methods using FDA-approved Pluronic F127 (PLU) block copolymer have garnered much attention for simultaneously forming and stabilizing Au nanoparticles (AuNPs). Given the remarkable properties of AuNPs for usage in various fields, especially in biomedicine, we performed a systematic study to synthesize AuNP-PLU nanocomposites under optimized conditions using UV irradiation for accelerating the reaction. The use of UV irradiation at 254 nm resulted in several advantages over the control method conducted under ambient light (control). The AuNP-PLU-UV nanocomposite was produced six times faster, lasting 10 min, and exhibited lower size dispersion than the control. A set of experimental techniques was applied to determine the structure and morphology of the produced nanocomposites as affected by the UV irradiation. The MTT assay was conducted to estimate IC50 values of AuNP-PLU-UV in NIH 3T3 mouse embryonic fibroblasts, and the results suggest that the sample is more compatible with cells than control samples. Afterward, in vivo maternal and fetal toxicity assays were performed in rats to evaluate the effect of AuNP-PLU-UV formulation during pregnancy. Under the tested conditions, the treatment was found to be safe for the mother and fetus. As a proof of concept or application, the synthesized Au:PLU were tested as contrast agents with an X-ray computed tomography scan (X-ray CT).

## 1. Introduction

Gold nanoparticles (AuNPs) display unique physicochemical properties, mainly related to the localized surface plasmon resonance (LSPR) phenomenon and following applications [1,2]. For multiple purposes, they are engineered into highly stable colloidal suspensions, and using different surface functionalization procedures, they show biocompatibility and colloidal stability in physiological conditions and in vivo assays [3,4]. Consequently, this nanomaterial has recently found meaningful applications in cell imaging and therapeutic processes in addition to different types of optoelectronic devices such as biosensors, solar cells, and surface-enhanced Raman spectroscopy, as well as in photocatalysis [5,6,7,8,9,10,11,12]. In the biomedical field, AuNPs have been used in electrochemical dopamine sensors [13] and intensified in early diagnosis of Parkinson’s disease [14]. New approaches to the fabrication of sensing platforms have been proposed, for instance, combining plasmonically active waveguides with microfluidics [15] or using single plasmonic nanoparticles as ultrasensitive sensors [16]. Concerning therapeutic uses, studies suggested AuNP systems for the controlled delivery of anticancer agents, resulting in enhanced antitumor activity with negligible toxicity to major organs [17]. Despite this, there is a need to thoroughly investigate the potential toxicological effects of AuNPs during pregnancy, particularly given their ability to cross the placental barrier and enter fetal circulation [18,19]. Studies have suggested that the size, shape, surface charge, and surface coating of gold nanoparticles can impact their toxicity, and it is likely that the same factors will influence their potential effects during pregnancy [19]. In addition, the route of administration may also be an important consideration, as the risks associated with intravenous administration may differ from those associated with topical or oral administration. Thereby, it is necessary to know all their possible toxicological effects [20] in order to harness all the advantages offered by this nanomaterial when delivering therapeutic formulations.

Regarding the light–matter interaction, the LSPR phenomenon in metal nanoparticles (NPs) is based on the resonance condition achieved between the frequencies for the collective oscillation of conduction band electrons in the NPs and the incident electromagnetic radiation. In the resonance condition, the wavelength of incident light is about ten times greater than the size of the NPs when the maximum LSPR extinction is reached. The resulting and very active LSPR band leads to unique scattering and absorption spectra for the NPs. In terms of NP structure, the resonance frequency depends on their size, shape, aggregation extent, surface chemistry, dielectric properties of the surrounding medium, and the level of interparticle interactions.

Among synthetic/engineering methods for producing AuNP with tailored characteristics, chemical methods have been dominant so far, enabling the production of a variety of nanostructures in sizeable amounts [21,22,23]. In addition, new approaches have been developed such as the novel high-throughput (HTP) synthetic platform for AuNPs, consisting of an HTP centrifugal microfluidic device and a portable automatic workstation. [24], sodium glutamate and sodium dodecyl sulfate as reducing and stabilizing agents [25], using the block copolymer template approach [26], and continuous in-flight synthesis [27]. Eco-friendly approaches have also been explored, for example, using natural extracts [28], biocompatible polymers [29], or light of different wavelengths [30] as reducing agents.

A particular type of chemical synthesis, which is the subject of the present contribution, is that performed at room temperature by simply mixing aqueous chloroauric acid (HAuCl_4_) and FDA (Food and Drug Administration)-approved block copolymers displaying a triblock PEO–PPO–PEO structure PEO: poly(ethylene oxide); PPO: poly(propylene oxide). Commercially available under the name Pluronic, one of its several grades, F-127 was used in this study These copolymers are water soluble and work simultaneously as reducing and stabilizer agents [29]. The main advantage of such a method is that colloidal stabilization is achieved simultaneously with NP formation and no other reducing agent is required. The dynamics of the redox reactions and the size and size distribution of the produced AuNPs are influenced by the Au:copolymer stoichiometry and the copolymer molecular weight/architecture. Indeed, these variables can be adjusted to produce AuNPs with controlled size and distribution. Isothermal calorimetry titration performed in a subsequent study revealed that during the formation of F127:Au nanocomposites, at a very low F127:Au molar ratio (below 0.05), the association between tetrachloroaurate anion (AuCl_4_^−^) and F127 is the prevailing event (exothermic process). However, at a larger F127:Au (0.05 to 0.12), particle growth becomes dominant when the Au^0^ nuclei and poly(propylene oxide) block interact by entropic hydrophobic forces [31]. In parallel, cyclic voltammetry indicated that complete reduction of Au^3+^ occurs only above the critical micellar concentration (cmc) (~0.2–0.8 wt%) of F127 [31]. Another study suggested that the size and polydispersity of these AuNPs depend almost exclusively on the copolymer concentration [32].

A recent detailed study on the formation of AuNPs as a result of the reduction of chloroauric acid with Pluronic block copolymers [33] demonstrated that in the course of this reaction, the copolymer chains undergo oxidation, although the oxidation of the polymer does not go all the way down to carboxylic acids. Analysis of the soluble products suggested that the copolymer undergoes partial degradation, preferentially at the PPO sites, with cleavage of the C–O bonds. Although the purified AuNPs contained organic components (13%, *m*/*m*), no PPO groups were observed in them, suggesting that PPO chains or intact Pluronic molecules are not adsorbed on the particle surface. Interestingly, the analysis of soluble products revealed the presence of the CH_3_ group of PPO. The product analysis also indicated the presence of newly formed OH groups both in the purified AuNPs and in the soluble products. The reaction involves the formation of free radicals and hydroperoxides, which depends on the oxygen concentration. The purified nanoparticles contain organic components but can be fully separated from the excess of the copolymer. Various reaction parameters such as pH, temperature, sodium chloride addition, and the concentration of the reactants affect the rate of the reaction and the yield and morphology of the resulting AuNPs [33]. A further step to optimize this synthesis process would be the implementation of UV irradiation to accelerate the synthesis and to control shape and physicochemical properties of the produced NPs [34,35,36,37,38]. The UV irradiation generates a photoexcited species ([AuCl_4_]^−^*) that undergoes successive disproportionation and reduction reactions leading to the formation of metallic gold atoms and the initial formation of gold nanoparticles [37]. This reduction process is faster than that promoted by the block copolymer alone, which mainly functions as a template for the formation of the nanoparticles. As a result of these different reaction dynamics, better control of the size distribution of the nanoparticles can be achieved.

Aiming at optimizing this eco-friendly, one-step synthesis method, by simply mixing aqueous chloroauric acid (HAuCl_4_) and PLU, here we report a systematic study involving the synthesis of Au:PLU nanocomposites assisted with UV irradiation. We found that the most important benefits of using UV irradiation 254 nm/16 W were that an Au:PLU nanocomposite of a specific formulation is produced about six times faster and with lower size dispersion than that made under ambient light. Those are great advantages since they offer a more cost-effective way to fabricate AuNPs with tailored properties to enable their use in different fields, especially in the biomedical field. A set of experimental techniques (UV-vis, Fourier Transform Infrared (FTIR) spectroscopies, thermogravimetry (TGA), transmission electron microscopy (TEM), measurement of the sedimentation coefficient using analytical ultracentrifugation (SV-AUC), and cyclic voltammetry) was performed to determine the structure and morphology of the nanocomposites as affected by UV irradiation. Then, the biological properties of the Au:PLU nanocomposites were assessed using in vitro cytotoxicity and in vivo maternal and fetal toxicity assays in rats. As a proof of concept or application, the Au:PLU synthesized in the absence/presence of UV irradiation were tested as contrast agents in an X-ray computed tomography scan (X-ray CT).

## 2. Materials and Methods

### 2.1. Materials

Pluronic block copolymer F-127 (PLU, MW 12,600 g/mol), chloroauric acid trihydrate (HAuCl_4_.3H_2_O) 99%, and dimethyl sulfoxide (DMSO) were purchased from Sigma–Aldrich (St. Louis, MO, USA). Analytical grade HCl 36%, HNO_3_ 65%, and KCl 99% were purchased from Vetec, Duque de Caxias-RJ, Brazil. All chemicals were used as received. All experimental procedures, including solution preparations and glassware cleaning, were performed with ultra-pure water (resistivity: 18 MΩ/cm) supplied by a Millipore Milli-Q water purification system. UV-assisted synthesis of AuNP-PLU nanocomposites was carried out in borosilicate beakers (5 mL), previously cleaned with aqua regia solution (HCl/HNO_3_, 3:1, *v*/*v*) and rinsed properly with ultrapure water.

### 2.2. UV-Assisted Synthesis of AuNP-PLU Colloidal Nanocomposites

**Figure 1:**
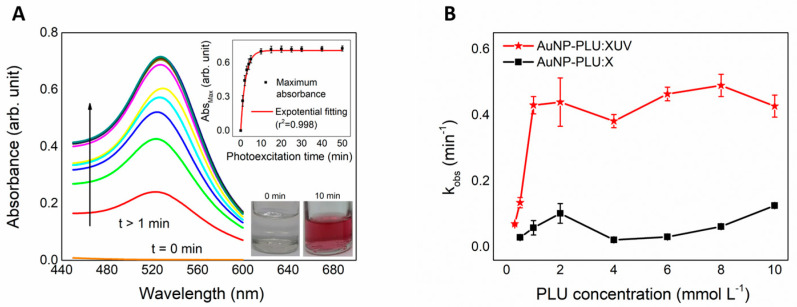
(**A**) UV-vis spectra obtained from monitoring the AuNP-PLU:2.0UV formation from the precursor mixture (HAuCl4 + PLU) at t = 0 and after 10 min. The formation of the AuNP-PLU:2.0UV nanocomposite is observed after 10 min. The lower and upper insets show digital snapshots of the reaction mixture at t = 0 min and t = 10 min and changes in the maximum absorption for AuNP-PLU:2.0UV of the plasmonic band during the photoexcitation time, respectively. (**B**) The observed rate constant of AuNP-PLU:2.0UV and AuNP-PLU:2.0 by the PLU concentration used in the synthesis process, as indicated.

Precursor aqueous solutions of HAuCl_4_ and PLU were prepared at room temperature with magnetic stirring. Colloidal AuNP-PLU were synthesized following the standard protocol as reported in [31] using gentle hand mixing of PLU and HAuCl_4_ aqueous solutions at proper compositions (10:1 *v*/*v*) and then leaving them to rest. Additionally, part of the reaction mixture was submitted to UV irradiation (254 nm, 16 W, 25 °C) inside a lab-made chamber as reported elsewhere [37]. Two sets of AuNP-PLU samples were prepared, with either fixed or varied concentrations of HAuCl_4_ and PLU, as described in Table 1. In group 1, samples were prepared with a fixed 2 mmol L^−1^ HAuCl_4_ concentration and PLU concentrations (X) varied between 0.1 and 10 mmol L^−1^. Samples labeled “UV” denote the reaction performed under UV 254 nm irradiation. This range of PLU concentrations comprises values below and above its critical micellar concentration (CMC), which is 0.6 mmol L^−1^ [39]. In group 2, the PLU concentration was kept fixed at 2.0 mmol L^−1^ to ensure the micellar structure, whereas the HAuCl_4_ concentration (Y) was varied between 1.0 and 4.0 mmol L^−1^, resulting in samples labeled YUV:AuNP-PLU. Control samples were prepared in the same way but under ambient light. The UV-assisted syntheses were carried out for different periods of time, up to 50 min, while the control sample was carried out for up to 300 min. Nonetheless, the reaction reached completion at about 10 min for most of the AuNP-PLU compositions, when it could be detected a color change in the reaction mixture from yellow to pink (Figure 1A). The UV-vis spectra precursors and control samples can be found in Supplementary Figure S1. After syntheses, the samples were transferred to common Eppendorf tubes (2 mL) and centrifuged (Mikro 22R, HETTICH Zentrifugen, Tuttlingen, Germany) at 12,000 rpm for 120 min at 10 °C to remove the excess PLU. In order to conduct Raman characterization and thermogravimetric analysis (TGA), solid samples of AuNP-PLU were obtained using extensive centrifugation and lyophilization as previously described [31] The obtained powder sample was labeled s-AuNP-PLU:XUV.

### 2.3. Structural and Morphological Characterizations

Ambient light and UV-assisted syntheses were continuously monitored, ex situ, using UV-vis spectroscopy in the range of 185–900 nm, at 0.1 nm resolution, and a scan rate of 600 nm/min (0.1 s integration by 1 nm) the spectrophotometer (Shimadzu UV 2600, Shimadzu Europe, Duisburg, Germany) and quartz cuvette of 1.0 cm optical path. UV-vis spectra were recorded at different time intervals until the absorbance of the LSPR band reached a plateau. FTIR spectra were registered using the attenuated total reflectance (ATR) method (Novertex 70, Bruker Corporation, Billerica, MA, USA), with a spectral resolution of 4 cm^−1^ and 1 scan/min for 27 min. An aliquot of 10 µL of the colloidal suspensions AuNP-PLU:2.0UV, AuNP-PLU:2.0 (used as control), or plain PLU solution was dropped onto the ATR crystal and left to dry. Afterward, successive spectra were recorded, monitoring the water’s hydroxyl stretching band (ν O–H), in order to confirm the sample was dried before registering representative spectra of samples.

Thermogravimetric analysis (TGA) curves were registered with a DTG60H (Shimadzu Corporation, Kyoto, Japan) system from 25 to 600 °C with a heating rate of 10 °C/min in a N_2_ atmosphere with a flux rate of 30 mL/min in an aluminum sample holder. This equipment provides measurements with an uncertainty of 0.5%/weight.

The morphology of the AuNP-PLU samples was observed using transmission electron microscopy (TEM) carried out with a JEOL 2100 microscope. The mean diameter (DTEM) and polydispersity index (σ) were determined from size histograms fitted with a log-normal distribution function. The diameters of approximately 1000 particles, measured with the aid of the Image J software in different TEM images, were used to build the histograms. In addition, the hydrodynamic diameter and zeta potential of colloidal samples were determined using dynamic light scattering and electrophoresis mobility, respectively, measured with a Zetasizer Nano ZS Malvern Instruments, UK. The analyses were performed at a scattering angle of 173° at 25 °C using a 4 mW He-Ne laser operating at 632.8 nm.

Cyclic voltammetry (CV) was used to estimate the amount of Au^+3^ ions that were not reduced during synthesis and thus qualitatively evaluate the efficiency of chemical/photochemical reduction. Voltammograms were recorded with a Metrohm potentiostat/galvanostat model Autolab PGSTAT 204 in a three-electrode configuration cell (Ag/AgCl reference electrode with saturated KCl, Pt wire as the counter electrode, and an indium-doped tin oxide (ITO) slide—0.60 cm^2^ active area—as the working electrode). The electrochemical cell was filled with the HAuCl_4_-PLU mixtures or AuNP-PLU samples containing KCl (0.1 mol L^−1^) as the supporting electrolyte. Experiments were carried out at 25 °C at 50 mV/s after purging the electrochemical cell with N_2_ for 5 min.

The particles produced using UV irradiation (AuNP-PLU:2.0UV) and using ambient light (AuNP-PLU:2.0) were investigated with sedimentation velocity analytical ultracentrifugation (SV-AUC) using a Proteome-Lab XL-A analytical ultracentrifuge equipped with a 4-hole titanium An-60 Ti rotor, cells with 12 mm path length, a double channel centerpiece, and quartz windows (Beckman Coulter, Brea, CA, USA). The assay was carried out at 2500 rpm at 20 °C in a vacuum system. Absorbance was measured at two wavelengths (260 and 530 nm) at scanning intervals for each sample reading of 2 min with 0.007 cm radial resolution. PLU absorbs at 260 nm, whereas AuNP-PLU absorbs at 530 nm. The collected radial scans were analyzed using the size distribution analysis ls-g(s) model with the SEDFIT v14.7 software using a resolution of 400, a sedimentation coefficient analysis interval of 0–10,000, and a confidence level of 0.951. The c(s) was calculated using the appropriate correction for the viscosity and density of water at 20 °C (S20,w) with the SEDFIT software.

### 2.4. Cell Viability Test

#### 2.4.1. Cell Culture

NIH-3T3 mouse embryonic fibroblast cells (ATCC^®^ CRL-1658TM) were cultured in Dulbecco’s Modified Eagle Medium (DMEM, Gibco^®^ Life Technologies, Ltd., Carlsbad, CA, USA) supplemented with 10% (*v*/*v*) fetal bovine serum (Gibco^®^ Invitrogen™, Waltham, MA, USA) and antibiotics (100 IU mL^−1^ penicillin and 100 μg mL^−1^ streptomycin-Sigma-Aldrich) at 37 °C in a 5% CO_2_ incubator.

#### 2.4.2. In Vitro Cytotoxicity Assay

The NIH-3T3 cells were seeded at a density of 5 × 103 cells per well in 96-well plates. After 24 h of incubation, 200 μL of the culture medium containing AuNP-PLU:2.0UV (0.22 to 3.4 μM AuNP; 0.03–0.41 mM PLU) were added to the wells for 15 min or 24 h. Following 15 min exposure, cells were rinsed twice with phosphate buffer saline (PBS pH 7.4), and then the culture conditions were reestablished for 24 h. The potential cytotoxic effect was determined using a (4,5-dimethylthiazol-2-yl)-2,5-diphenyltetrazolium bromide (MTT) (Waltham, MA, USA) assay. Cells were incubated with MTT solution (5 mg mL^−1^) for 2 h, and the colorimetric product, formazan crystals, was solubilized in DMSO. The absorbance values were measured with a spectrophotometer (Molecular Devices SpectraMax M2^®^, Silicon Valley, CA, USA) at 595 nm. The raw data were normalized to the control data (cells treated with culture medium containing PBS, PBS replaced AuNP-PLU:2.0UV). The IC50 value (half-maximal inhibitory concentration) was calculated using a nonlinear regression dose–response analysis with AuNP concentration in logarithm. Results were expressed as mean ± standard deviation (SD) of triplicate determinations from three independent experiments (n = 9/concentration). The AuNP-PLU:2.0 (0.05 to 0.86 μM AuNP; 0.03–0.48 mM PLU) and PLU solutions were tested under the same conditions for comparison purposes.

### 2.5. In Vivo Maternal and Fetal Toxicity of AuNP-PLU:2.0UV in Rats

Adult female (180–200 g) and male (220–240 g) Wistar rats were maintained in cages for three rats with autoclaved wood chips under standard laboratory conditions (23 ± 2 °C, humidity 50 ± 10%, 12 h light/dark cycle), with pelleted food and tap water provided ad libitum. The local Experimental Ethical Committee for Animal Research approved procedures and animal handling protocols used in this study (Protocol Number: 23108.022668/2019-23).

After two weeks of acclimation in the vivarium of the Laboratory of System Physiology and Reproductive Toxicology, Federal University of Mato Grosso (UFMT), all female rats were mated overnight with male rats. Gestational day zero (D0) was recorded and assigned the following morning when spermatozoa were found in the vagina smear [40]. The pregnant rats were randomly distributed using computer random numbers into two experimental groups (n = 12 animals/group): Control = pregnant rats treated with water and Treated = pregnant rats treated with AuNP-PLU:2.0UV. The rats were treated with AuNP-PLU:2.0UV or vehicle (water) in the morning with the intragastric route (gavage) during pregnancy (from gestational day 0 to 21). The dosage selection of AuNP-PLU (137.5 µg/kg) was made according to the previously reported dose [4], which in humans is equivalent to 22 µg/kg based on the body surface area (BSA) [41].

On days 0, 7, 14, and 21 of pregnancy (at 9 a.m.), the maternal body weights, food, and water consumption were determined. On day 21 of pregnancy, the rats were anesthetized using sodium pentobarbital (Thiopentax^®^-120 mg/kg). Then, they were submitted to laparotomy for exposure to uterine horns. The gravid uterus was, withdrawn, weighed, and dissected. The percentage of embryonic loss before and after implantation was calculated [42]. Fetuses and placentas were weighed. Fetuses were then classified by body weight [43] and evaluated to determine the presence of external anomalies. After external analysis, half of the fetuses were fixed in Bouin’s fluid, and serial sections were prepared as described by Wilson [44] for visceral examination. The remaining fetuses were prepared for an examination of skeletons using the staining procedure of Staples and Schnell [45]. In addition to the skeletal analyses, counting of ossification sites was performed to determine the degree of fetal development [46,47].

The Student’s t-test was performed to evaluate mean values, whereas the Fisher Exact test was conducted to compare percentages. *p* < 0.05 was applied and considered as the limit of statistical significance.

### 2.6. X-ray Computed Tomography Scan

CT images were performed at the Laboratório de Caracterização Tecnológica, Escola Politécnica, Universidade de São Paulo-SP, Brazil. Liquid samples containing the synthesized nanocomposites were stored in Eppendorf and analyzed in batches containing 6 samples using the 3D X-ray microscope: Zeiss Xradia Versa XRM-510 equipment, for beam energies of 80, 100, 120, and 140 KV. The attenuation coefficients of air and deionized water were used for calibration. The AuNP-PLU:2.0UV with Au of 1.23 mg/mL sample was compared with Optiray@320_1 (Liebel-Flarsheim Company LLC, Raleigh, NC, USA (EUA) containing I at the same concentration (I = 1.23 mg/mL) and AuNP-PLU: 2.0 containing a Au concentration of 1.09 mg/mL was compared with Optiray@320_2 with I at the same concentration (I = 1.09 mg/mL).

## 3. Results and Discussion

### 3.1. Effect of the UV Light on the Features of Synthesized AuNP-PLU

#### 3.1.1. Kinetics of UV-Assisted Synthesis of AuNP-PLU

The formation of the AuNP-PLU NC under UV light was monitored using UV-vis spectroscopy with the evolution of the typical LSPR band of AuNPs. An overview of the formation of AuNP-PLU:2.0UV, chosen as the reference sample, is provided in Figure 1. In Figure 1A, the UV-vis spectra recorded at different time intervals display the LSPR band typical of AuNPs from the very beginning of the reaction. The LSPR band is centered at 526 nm for t = 10 min. The inset in Figure 1A shows digital snapshots of the reaction mixture at t = 0 min and t = 10 min. After 10 min of reaction, the typical pink color of AuNPs is clearly seen. Otherwise, for the reaction carried out under ambient light, the formation of AuNPs is visually detected solely after 60 min (as observed in ref. [32], where the LSPR band is centered at 536 nm), thus indicating that UV irradiation accelerates the reaction and the LSPR band of AuNP-PLU:2.0UV is blue-shifted in comparison to the sample AuNP-PLU:2.0. Indeed, Supplementary Figure S2 displays isotherms for the formation of AuNP-PLU under ambient light (AuNP-PLU:2.0) (data from ref. [32]) and UV irradiation (AuNP-PLU:2.0UV). Although both isotherms show an asymptotic behavior, it is noted that the reaction is much faster in the presence of UV irradiation, which reaches equilibrium in about 10 min, in contrast to the reaction carried out under ambient light, which reaches equilibrium in about 60 min. Experimental data were further fitted with a first-order kinetics model as expressed by Equation (1):

(1) $${Abs}_{max}={Abs}_{\infty }\left(1-{exp}^{-k_{obs}t}\right)$$

In Equation (1), *Abs_max_* is the maximum LSPR absorbance (in arbitrary units) in the reaction time *t* (expressed in min), *Abs_∞_* is the absorbance of the LSPR band at an infinite time of reaction (in arbitrary units), *k_obs_* is the observed rate constant (in min^−1^), and *t* is the reaction time (in min). As shown in Figure 1B, *k_obs_* becomes independent of the PLU concentration as the cmc condition is reached for both reaction conditions (ambient light and UV irradiated). Nonetheless, *k_obs_* is about four times larger under UV irradiation.

#### 3.1.2. Evaluation of the Yield of AuNP-PLU-UV Production

**Figure 2:**
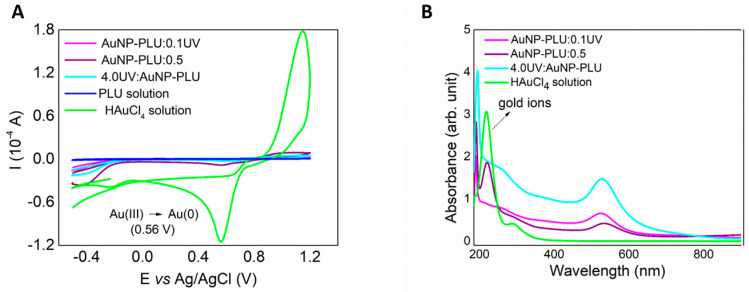
Cyclic voltammograms of the HAuCl4 solution at a concentration of 2.0 mmol L^−1^, and the AuNP-PLU:0.1UV (lowest PLU concentration), 4.0UV:AuNP-PLU NCs, and AuNP-PLU:0.5 control sample, as indicated. (**B**) UV-vis spectra of the same samples displayed in (**A**), as indicated.

Cyclic voltammetry (CV) was used to further investigate the effect of the UV irradiation by measuring the amount Au^+3^ ions left over during reduction by PLU. This experiment was conducted with the sample AuNP-PLU:0.1UV, which was produced with the lowest PLU concentration, as well as with the sample 4.0UV:AuNP-PLU, which was produced with the largest HAuCl_4_ concentration. In addition, the experiment also used the sample AuNP-PLU:0.5, which was prepared under ambient light with the smallest PLU concentration, but sufficient to produce sizeable amounts of AuNPs. As shown in Figure 2A, the voltammogram (green curve) of the plain HAuCl4 solution clearly shows the Au^0^/Au^3+^ redox pair at +1.0 V and +0.56 V, respectively, plus the cathodic wave for Au^1+^/Au^0^ at –0.2 V. The voltammogram of plain PLU shows a subtle anodic event at +0.11 V. Indeed, this is sufficient to reduce Au^3+^ to Au^0^, which occurs at a much higher potential, as discussed in a previous contribution [32]. On the other hand, these electrochemical signals almost disappear in the voltammograms of the nanocomposites as a result of the Au^3+^ reduction. Nonetheless, the sample AuNP-PLU:0.5 still has a considerable residual amount of Au^3+^ (about 7 wt%), whereas in AuNP-PLU:0.1UV, the nanocomposite produced under UV irradiation, this amount is only 0.3 wt%, even though this sample was produced using a 5-fold lower PLU concentration. It is worth noting that the sample 4.0UV:AuNP:PLU, which was produced in a less favorable composition with an excess of HAuCl_4_, presented only 1.5 wt% of unreduced gold ions. These observations are corroborated further by the UV-vis spectra of these samples, as shown in Figure 2B. In particular, the absorption of AuCl_4_^-^ species at 220 nm is only detectable in the spectra of the plain HAuCl_4_ solution and AuNP:PLU:0.5, which were not submitted to UV irradiation. In summary, the implementation of UV irradiation not only accelerated the reaction performed under ambient light but also increased its yield, even at a very low PLU concentration (AuNP-PLU:0.1UV).

The percentual of PLU in the nanocomposites was estimated using TGA curves, shown in Supplementary Figure S3. The TGA curves of the plain PLU, AuNP-PLU:2.0UV, and AuNP-PLU:2.0 are very similar showing a single mass loss event around 400 °C, which is regarded as the degradation of PLU. According to the derivative TGA, however, the degradation of PLU occurs slightly below this temperature in the nanocomposites as a result of the oxidation of PLU by Au^3+^. The estimated concentration of PLU in AuNP-PLU:2.0UV and AuNP-PLU:2.0 is approximately 20 mg/mL (1.58 mmol L^−1^) and 24 mg/mL (1.90 mmol L^−1^), respectively. In addition, the atomic absorption analysis performed on AuNP-PLU:2.0UV and AuNP-PLU:2.0 samples gives a gold concentration of 3.12 mmol L^−1^ and 2.77 mmol L^−1^, respectively. These results corroborate those achieved by cyclic voltammetry, which also suggested that the UV-assisted synthesis process has a higher yield than that conducted under ambient light in the same compositional conditions.

#### 3.1.3. Morphology of AuNP-PLU

**Figure 3:**
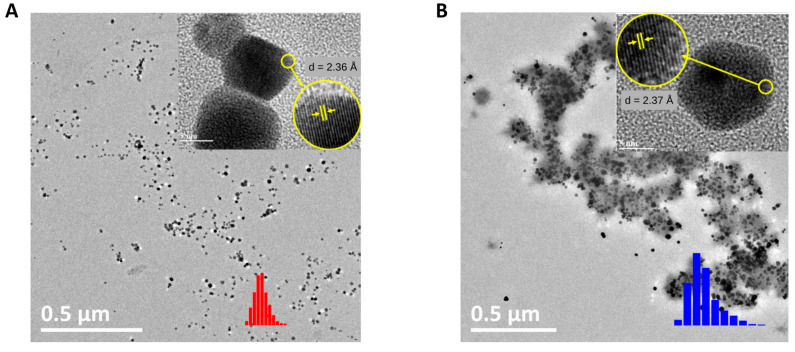
TEM micrographs of the AuNP-PLU:2.0UV sample (**A**) and of the AuNP-PLU:2.0 control (**B**) both with a scale bar of 0.5 µm and 5 nm in the respective insets. The upper insets depict a crystalline plane {111} of gold nanoparticles; the lower insets (red and blue) show the nanoparticle diameter histograms as vertical bars, as indicated. Data of (**B**) were reproduced with permission [31]. Copyright 2018, Elsevier B. V.

The morphology of the nanocomposite and the mean size of the AuNP cores were evaluated using TEM. Typical TEM micrographs of the AuNP-PLU:2.0UV and AuNP-PLU:2.0 samples are provided in Figure 3A and Figure 3B, respectively. Both samples are composed mainly of spheric-like nanoparticles. The high-resolution micrographs shown in the inset also reveal a high crystalline appearance with regularly spaced crystallographic planes. The interplanar distance in each sample was assessed using ImageJ software as follows: 2.36 Å (AuNP:PLU:2.0) and 2.37 Å (AuNP:PLU:2.0UV). These values are comparable to that provided by the Joint Committee on Powder Diffraction Standards-crystallographic plugs for the fcc structure of gold, which corresponds to the {111} family planes.

Particle size histograms were built with sizes measured in TEM micrographs and fitted with a log-normal distribution function give the following diameter (D_TEM_) and standard diameter deviation (σ) attained from nanoparticle diameter histogram (vertical bars) fitted with a log-normal distribution function, as displayed in Supplementary Figure S4. The results were 12.2 ± 0.2 (σ = 0.2 ± 0.02) and 18.5 ± 0.2 nm (σ = 0.3 ± 0.27) for AuNp-PLU:2.0UV and AuNP-PLU:2.0 [31], respectively. It is noticeable that AuNPs prepared with UV irradiation are smaller with a standard diameter deviation lower than the control AuNp-PLU:2.0. These findings show an improvement in the morphology of AuNPs produced under UV irradiation. Furthermore, a thin polymeric coating around the UV-synthesized AuNPs is seen in the TEM images (Figure 3A), while AuNPs control samples are enfolded by the polymer (Figure 3B). Although the estimated concentration of PLU in AuNP-PLU:2.0UV is only 20% less than in AuNP-PLU:2.0, it is intriguing that only a thin PLU coating was seen in the micrographs of the former. This observation may be associated with the higher yield of AuNPs when using UV light, but it could also be related to a more extensive degradation/oxidation of PLU under UV light. These findings suggest an improvement in the morphology of AuNPs produced under UV irradiation.

The concentration of AuNPs present in the NCs was estimated considering the data of the mean diameter D_TEMt_ and the gold concentration obtained with atomic absorption measurements. The values found were 13.6 and 3.4 µmol L^−1^ for the AuNP-PLU-2.0UV and AuNP-PLU:2.0 samples, respectively. Considering those values as the amount of PLU being adsorbed by AuNPs, the molar ratios given by the amount of PLU by AuNPs [PLU/AuNPs] for AuNP-PLU:2.0UV and AuNP-PLU:2.0 are 116 and 559, respectively. These results indicate that the amount of PLU by AuNP in the control sample is approximately 4-fold greater than that in AuNP-PLU:2.0UV, which agrees with the TEM micrograph shown in Figure 3B.

According to the scattered intensity distribution data obtained with DLS measurements (Supplementary Figure S5), the average hydrodynamic diameter of AuNP-PLU:2.0UV and AuNPPLU:2.0 is 37.2 ± 2.2 nm and 50.2 ± 0.4 nm, respectively, with a PDI of 0.2. These findings indicated that the incidence of UV irradiation not only accelerates the reaction, but it also decreases the size of the AuNP cores and the hydrodynamic size produced by nanocomposites.

**Figure 4:**
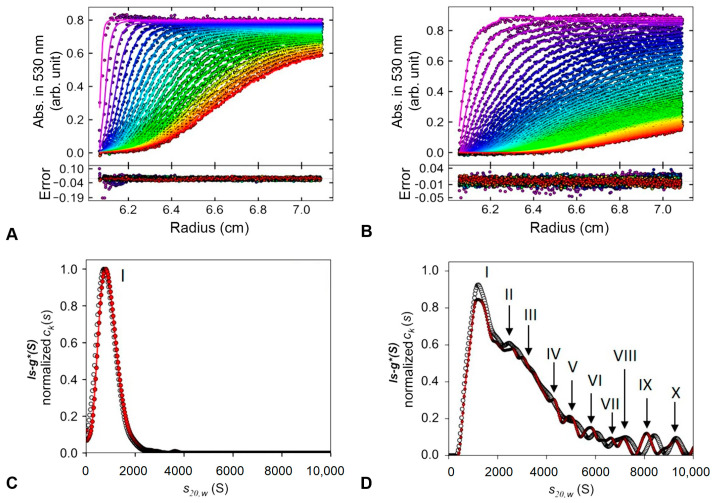
Sedimentation velocity analytical ultracentrifugation assay. Typical raw sedimentation profiles of absorbance at 530 nm versus cell radius for the particles present in the NCs are shown in (**A**,**B**). Residual plot produced using SEDFIT v14.7 software showing the fitting goodness. The lower panels show the apparent distributions of the sedimentation coefficient Is−g* (S) at 260 nm (black) and 530 nm (red) obtained for AuNP-PLU:2.0UV (**C**) and control AuNP-PLU:2.0 (**D**). The peaks correspond to different populations of nanoparticles and are indicated with Roman numerals.

The size distribution of the NCs produced with UV-assisted synthesis and the control using environment light was further analyzed using the apparent sedimentation coefficient distribution (ls-g*(s)) in an SV-AUC assay, as described in the experimental section. This approach is used without any knowledge of the partial specific volumes of the sedimentation species, according to those previously described. Figure 4 displays the results for AuNP-PLU:2.0UV and AuNP-PLU:2.0.

When AuNP-PLU:2.0UV (Figure 4A) is subjected to a high-field centrifuge, uniform sedimentation of the particles is observed with an exponential curve behavior that reaches a concentration plateau (abs. ∼0.8). The best fit of the curve in Figure 4A was attained with a mathematical model based on the Lamm, which describes the spatial and temporal behavior of the concentration considering the sedimentation by mass transport by diffusion [48]. Using the ls-g*(s) procedure, fair RMS deviation values (<0.007) were obtained for both data sets. In the case of ls-g*(s) sedimentation profiles at 530 nm for photoexcited AuNP-PLU:2.0UV, a single peak (Figure 4C) with sedimentation coefficient S20,w of 947.10S was observed, indicating a homogeneous population of nanoparticles (Figure 4D and Table 2).

However, for AuNP-PLU:2.0, which was produced under ambient light (Figure 4B), faster sedimentation is observed, which may be associated with the presence of larger particles. Similar to our previous report [31], the ls-g*(s) sedimentation distribution profiles at 260 nm and 530 nm for this NC showed the highest and broadest peak with smaller and less abundant ones, probably formed by heterogeneous particle populations according to the distribution analyses.

The main particle population measured at 530 nm corresponds to peak I (43.2%) with a sedimentation coefficient S20,w of 1451.17S, while the next two populations that contribute significantly are those related to peak II (13.3%) and III (20.04%) with 2613.09S and 3477.32S, respectively (Figure 4D and Table 2). Other peaks with less than 5% in the distribution analyzed are also detected, suggesting a small content of other particle sizes. A control experiment performed with plain PLU showed that the same conditions of ultracentrifugation are unable to sediment the polymer during the experimentation time. The similarity between the ls-g*(S) distribution versus S20,w at 260 and 530 nm of the samples suggests that all particles detected are formed by complexes of gold and polymer. The absence of polymeric aggregates without gold in its structure indicates that the presence of gold or UV irradiation, by itself, does not induce spontaneous aggregation of the polymer.

The SV-AUC results are in agreement with those found using transmission electron microscopy (TEM), presented in Figure 3, where the sample synthesized with UV photoexcitation (AuNP-PLU:2.0UV) presented a narrower size distribution. Indeed, the TEM and SV-AUC characterizations show that implementation of UV irradiation improves the reaction by producing samples with a more controlled size and size distribution.

#### 3.1.4. The Role of UV Light and PLU on the Formation of AuNP-PLU

**Figure 5:**
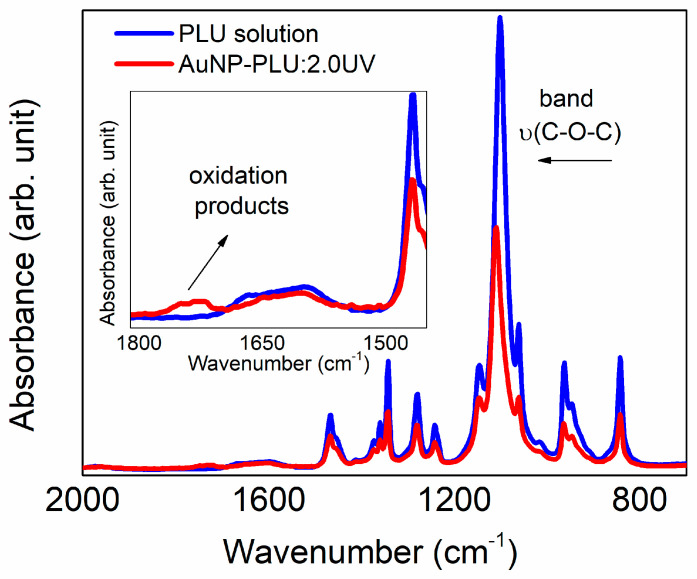
ATR FTIR spectra obtained for aqueous solutions of F127 at a concentration of 2.0 mmol L^−1^ and the AuNP-PLU:2.0UV sample after 10 min of photoexcitation. Inset: enlarged view of the region close to 1700 cm^−1^.

The results of the UV-vis and FTIR-ATR analyses presented in Figure 1 and Figure 5 showed that the PLU and HAuCl_4_ solutions do not show spectral variations when exposed to UV radiation. Thus, the formation of AuNP-PLU is surely related to the effect of irradiation in the reaction mixture (PLU + HAuCl_4_).

The structural features of nanocomposites were evaluated further using ATR FTIR and Raman spectroscopies. As shown in Figure 5, the ATR-FTIR spectrum of plain PLU after being submitted to UV irradiation for 10 min does not show any signs of additional oxidation or any conformational change, remaining identical to that not exposed to UV [31]. On the other hand, the spectrum of AuNP-PLU:2.0UV contains an additional broad band between 1700 and 1750 cm^−1^, which is ascribed to the C=O stretching in aldehydes, ketones, or carboxylic acids, thus confirming the oxidation of the polymer when irradiated in the presence of AuCl_4_^−^ ions. In addition, the ν(C-O-C) band is shifted to higher wavenumbers (1100 to 1110 cm^−1^). This shift could be related to the presence of a lower amount of PLU, as observed in the TGA analysis of AuNP-PLU:2.0UV, or changes in PLU molecular weight due to more extensive polymer degradation and oxidation in the UV light method.

It is known that the thermal reduction of AuCl_4_^−^ in the presence of an aqueous solution of various organic molecules creates AuNPs by the nucleation and growth processes [49]. Usually, the first step is the association of anionic Au^3+^ complexes with the organic molecules, followed by a multiple-step redox reaction that results in Au^0^ nuclei and oxidized organic molecules. In these cases, the size of AuNPs depends strongly on the rate of reduction of the Au^3+^ species; an increase in the rate of the redox reaction creates a large number of Au^0^ nuclei and decreases the number of oxidized gold species in the solution. The consequence is a more uniform growth of small AuNPs. The mechanism of AuNP formation under UV irradiation of a reaction mixture containing HAuCl_4_ and citric acid seems to be similar to the respective thermal mechanism, with the photoexcited citrate–AuCl_3_^−^ complex being responsible for the speeding up the formation of Au^0^ nuclei [49].

It is well established that when trivalent gold ions AuCl_4_^−^ are irradiated with UV-light, the reduction occurs in successive steps: (i) reduction into the unstable bivalent state Au^2+^; (ii) disproportionation of Au^2+^ into Au^3+^ and Au^1+^; and (iii) reduction of accumulated Au^1+^ ions to Au^0^ [35]. It is worthwhile to note that Au^1+^ is not easily reduced as long as Au^3+^ ions are present in high amounts, and it does not disproportionate because the reduction potential Au^1+^/Au^0^ is considerably negative (−1.4 V). Mallick et al. propose that photolysis of H_2_O (UV source dependent) generates both H and OH radicals that can react with organic molecules producing strong reducing molecular radicals, which are able to reduce Au^1+^ to Au^0^ [36]. Thus, it can be concluded that the presence of organic radicals is very likely to accelerate the formation of Au^0^ nuclei. In this regard, Sokolsky-Papkov and Kabanov studied the formation of AuNPs from HAuCl_4_ in the presence of Pluronic F127, and they found a strong relation between the presence of oxygen reactive species (i.e., superoxide, hydroxyl radicals, hydrogen peroxide), which were formed by the decomposition of the hydroperoxides initially present in Pluronic, and the characteristics of AuNPs [34]. Simply stated, as the amount of oxygen-reactive species increases, the size and polydispersity of AuNPs reduce. Further, they concluded that Pluronic undergoes oxidation/degradation with the formation of lower molecular mass alcohols. Although this detailed study was performed without UV irradiation, it is reasonable to consider that these oxygen-reactive species play an important role in the formation of AuNPs from HAuCl_4_/PLU mixtures under UV light, and it may explain a great part of our results regarding reaction time, size, morphology, yield, and polydispersity of AuNps.

### 3.2. In Vitro Cytotoxicity Assay

**Figure 6:**
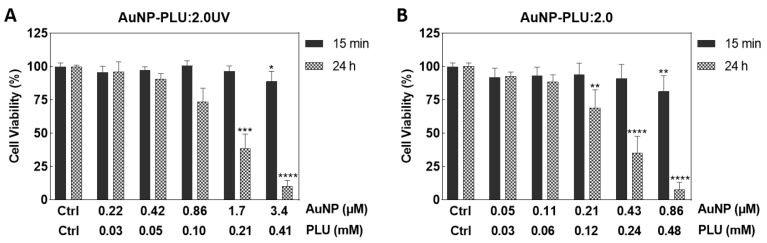
Effect of AuNP-PLU:2.0UV or AuNP-PLU:2.0 on viability of the NIH-3T3 cells determined using an MTT assay. Cells were incubated with AuNP-PLU:2.0UV (**A**) or AuNP-PLU:2.0 (**B**) for 15 min or 24 h. AuNP concentrations ranged from 0.22 to 3.4 μM in the AuNP-PLU:2.0UV sample and 0.05 to 0.86 μM in the AuNP-PLU:2.0 sample. Data are presented as mean ± standard deviation (SD) from three independent experiments. Differences among groups were determined using the Kruskal–Wallis test and Dunn’s multiple comparison post hoc test. Asterisks indicate significant differences compared to the respective control group: * *p* < 0.05, ** *p* < 0.01, *** *p* < 0.001, **** *p* < 0.0001.

Figure 6 shows the effect of concentration and exposure time of AuNP-PLU:2.0UV and AuNP-PLU:2.0 on the viability of NIH-3T3 cells. In the short term (15 min), the largest concentrations of AuNP-PLU:2.0UV and AuNP-PLU:2.0 caused a slight reduction in viability compared to controls, less than 20%. However, after 24 h of incubation, AuNP-PLU:2.0UV (at 1.7 µM) and 3.4 µM AuNP reduced respectively, about 62 and 90% of NIH-3T3 cells viability, compared to control group. Additionally, for the same dose (0.86 µM), AuNP-PLU:2.0UV was significantly less toxic (reduction of 26%) than AuNP-PLU:2.0 (reduction of 92%). The IC50 value estimated for AuNP-PLU:2.0UV was 1.29 µM (R^2^ = 0.7453), whereas the value for AuNP-PLU:2.0 was 0.32 µM (R^2^ = 0.7495). Cells treated only with PLU showed viability greater than 80% (Supplementary Figure S6).

According to these findings, AuNP-PLU:2.0UV nanocomposites are potential candidates for use as biocompatible carriers of drug and/or contrast agents. In general, information about the toxicity of NPs, particularly in vulnerable populations such as pregnant women and their fetuses, has lagged behind the development of new applications. AuNPs can cross the placental barrier and directly damage fetal tissues and/or interfere with proper placental development and function, creating a hostile gestational environment for fetal growth and development [50]. There is evidence that 3–4 nm AuNPs cross the human placental barrier in limited amounts and accumulate in the placental, depending on their size and surface modification [51]. To support the safety-by-design concept within nanotechnology research, we seek to understand if AuNP-PLU:2.0UV impacts maternal–fetal health using a model of pregnant rats.

### 3.3. In Vivo Maternal and Fetal Toxicity in Rats

While there may not be many studies specifically evaluating the maternal toxicity or teratogenicity of AuNPs during pregnancy, there is some evidence suggesting that these particles could have harmful effects [52,53,54]. Therefore, the toxicological effects of AuNPs for both mother and fetus were evaluated. Table 3 shows the maternal toxicological parameters, and no differences were noted among the groups in maternal weight, gravid uterus weight, food and water consumption, relative organ weight, and biochemical parameters. In an animal laboratory, factors such as body weight and water and feed consumption, as well as organ weight, are considered important parameters in the assessment of the systemic toxicity of a substance [55]. In addition, increased AST and ALT concentrations in blood are used as biochemical markers of hepatic tissue damage, and urea and creatinine are used to evaluate renal function [43]. Our data showed no differences in these parameters between groups, thus suggesting that treatment performed with AuNP-PLU:UV should not impart maternal toxicity.

**Figure 7:**
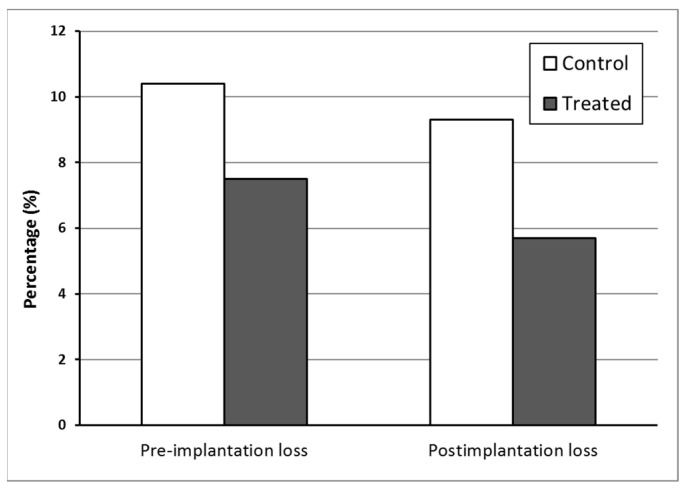
Percentage of losses of embryos before and after implantation at the term of pregnancy (DP21) for rats treated with water (Control) or AuNP (Treated) during pregnancy. *p* > 0.05 compared with the Control group (Fisher’s Exact test).

For the success of pregnancy, it is essential that, during the implantation period, the physiological and molecular processes are coordinated, involving close interactions between the uterus and the blastocyst [56]. The pre- and post-implantation loss rates did not differ statistically among the groups (Figure 7).

**Figure 8:**
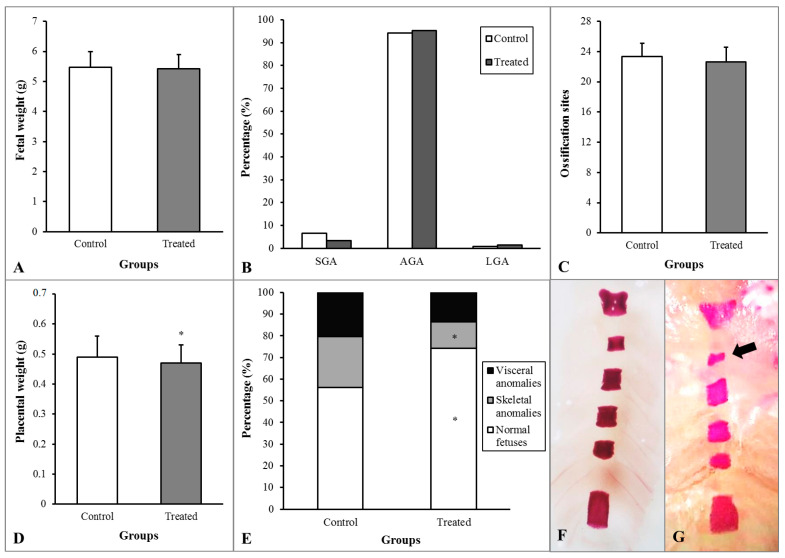
Fetal and placental development of rats treated with water (Control) or AuNP (Treated) during pregnancy. (**A**)—Fetal weight. (**B**)—Percentages of fetuses classified as small (SGA), adequate (AGA), or large (LGA) for gestational age. (**C**)—Ossification sites of fetuses. (**D**)—Placenta weight. (**E**)—Percentage of anomalies. (**F**)—Image of normal sternebra of rat fetuses. (**G**)—Incomplete ossification of sternebra (arrow). * *p* < 0.05 compared with the Control group (A,C,D—Student’s *t*-test; B,F—Fisher’s Exact test).

During pregnancy, exposure to toxic agents may lead to different effects ranging from functional or morphological changes, developmental delay, and anomalies to lethality in fetuses [57]. Figure 8 shows fetal and placental development. The rats that received AuNP presented a decrease in placenta weight and skeletal anomalies rate and an increase in normal (without anomalies) fetuses rate. Although the AuNP-PLU:UV treatment decreased placental weight, this modification did not affect fetal development, which showed unchanged fetal weight, weight classification, and ossification sites.

### 3.4. X-ray CT Scanner

X-ray computed tomography (CT) is an imaging technique used to uncover the interior features of a patient body in a three-dimensional view. The CT image stems from attenuation of the X-ray intensity by the body parts, in such a way that denser parts will attenuate more than softer ones. CT images can be produced from plain organs, but better resolution is only achieved using contrast agents, such as iodine and gadolinium. Indeed, greater atomic number elements will attenuate the incoming X-ray more [58].

**Figure 9:**
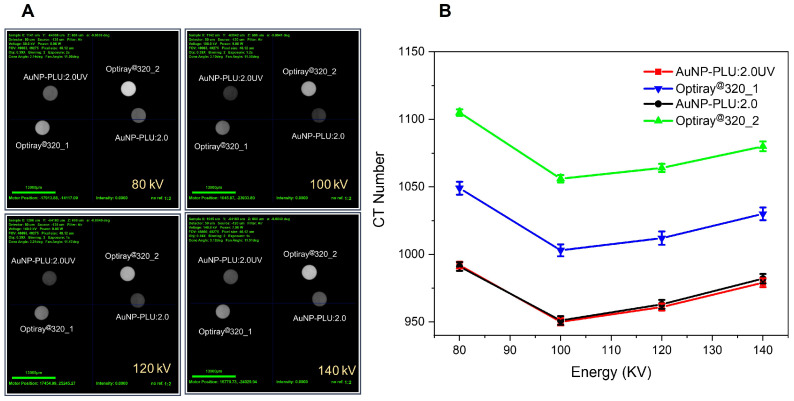
(**A**) CT images of AuNP-PLU:2.0UV with Au of 1.23 mg/mL compared with Optiray@320_1 containing I at the same concentration (I = 1.23 mg/mL), and AuNP-PLU:2.0 containing a Au concentration of 1.09 mg/mL compared with Optiray@320_2 with I at the same concentration (I = 1.09 mg/mL) attained for beam energies of 80, 100, 120, and 140 KV. (**B**) CT number attained for the same samples displayed in (**A**) as a function of beam energy.

Figure 9A shows CT images of AuNP-PLU: 2.0UV with Au of 1.23 mg/mL, compared with Optiray@320_1 containing I at the same concentration (I = 1.23 mg/mL), and AuNP-PLU: 2.0 containing Au concentration of 1.09 mg/mL, compared with Optiray@320_2 with I at the same concentration (I = 1.09 mg/mL) attained for beam energies of 80, 100, 120, and 140 KV. Figure 9B displays variations in the CT number with the beam energy for the commercial iodine contained contrast Optiray@320 and photochemically made Au-PLU:2.0UV. To compare, samples of both contrast agents were tested at the same concentration (expressed in mmol L^−1^ of iodine or gold). Data from AuNP-PLU:2.0UV containing a Au concentration of 1.23 mg/mL was compared with data from Optiray@320_1 with an I concentration of 1.23 mg/mL. Similarly, AuNP-PLU: 2.0 with a Au concentration of 1.09 mg/mL was compared with an Optiray@320_2 sample with an I concentration of 1.09 mg/mL.

As shown in Figure 9B, the Au-PLU samples provide CT contrast comparable to iodine for a same beam energy. Likewise, the CT number achieved with both agents is similar and decreases more or less linearly with the beam energy, as shown in Figure 9B. Furthermore, it is observed that the nanocomposite AuNP-PLU:2.0UV shows a closer CT number to its iodine control (Optiray@320_1) than the AuNP-PLU:2.0 when compared with Optiray@320_2. It is noteworthy that despite its established use, iodine as a contrast agent can promote unwanted side effects, ranging from a simple skin allergy to more acute effects including vascular permeation and renal toxicity [59]. Indeed, many patients cannot use iodine at all when submitted to CT procedures. Gadolinium is an alternative, although it provides lower contrast at comparable doses [60]. Recently, AuNPs were tested for this task and showed even better attenuation ability than iodine allied to longer circulation time, which enables for more accurate observation of vessels and tissues [61]. Furthermore, they can be designed with biocompatible and non-toxic surface coatings, which mitigate eventual side effects during administration [62]. Considering that the proposed photochemical method uses light instead of a reducing agent and that PLU is recognized for its biocompatibility and negligible toxicity, the present Au-PLU:2.0UV is a potential contrast agent for CT.

## 4. Conclusions

In this study we employed an UV-assisted synthesis protocol to produce biocompatible colloidal nanocomposites, comprising gold nanoparticles (AuNPs) and Pluronic F127 (PLU). Under optimized conditions, the proposed synthesis process produces AuNP-PLU nanocomposites at much faster rates (at least six times) than the process conducted under ambient light. Moreover, the synthesized nanoparticles display a controlled shape, narrower size distribution, and lower levels of PLU. Cytotoxicity assays demonstrate that the AuNP-PLU produced under UV irradiation is significantly less toxic than that produced under ambient light. In vivo maternal and fetal toxicity assays in rats reveal this nanocomposite is safe for both mother and fetus. As a proof of concept, we tested the nanocomposite as a contrast agent in X-ray computed tomography scans and found that it performs similarly to the commercially available iodine contrast agent Optiray@320. This is a promising feature, suggesting that AuNP-PLU synthesized under UV irradiation has high potential as a biocompatible nanocarrier and contrast agent.

## Figures and Tables

**Table 1:** Labeling of samples resulting from mixtures of aqueous solutions of precursors.

Samples	Concentration (mmol L^−1^)	
	PLU (*X*)	HAuCl_4_ (*Y*)
AuNP-PLU:0.1UV	0.1	2.0
AuNP-PLU:0.3UV	0.3	
AuNP-PLU:0.5UV	0.5	
AuNP-PLU:1.0UV	1.0	
AuNP-PLU:2.0UV	2.0	
AuNP-PLU:4.0UV	4.0	
AuNP-PLU:6.0UV	6.0	
AuNP-PLU:8.0UV	8.0	
AuNP-PLU:10.0UV	10.0	
1.0UV:AuNP-PLU	2.0	1.0
3.0UV:AuNP-PLU		3.0
4.0UV:AuNP-PLU		4.0

**Table 2:** Sedimentation parameters of the nanoparticles.

Synthesis Methods	Distribution at 530 nm			Distribution at 260 nm		
	ls-g* (S) Peaks	S20,w	Percentage Ratio (%)	ls-g* (S) Peaks	S20,w	Percentage Ratio (%)
**Environment light** **(Control)**	I	1451.17	43.21	I	1418.534	44.17
	II	2613.09	13.30	II	3555.02	43.80
	III	3477.32	20.04	III	6118.22	2.71
	IV	4403.81	5.24	IV	7145.20	3.26
	V	5030.07	4.98	V	8441.53	2.02
	VI	5811.97	3.67	VI	9364.58	1.69
	VII	6343.14	0.85			
	VIII	6681.68	1.44			
	IX	7198.76	1.44			
	X	8125.44	2.16			
**photoexcited synthesis (UV)**	I	947.10	99.36	I	857.78	99.15

**Table 3:** Maternal toxicological parameters of rats treated with water (Control) or AuNP-PLU:2.0UV (Treated) during the pregnancy.

	Groups	
	Control (n = 12)	Treated (n = 12)
**Weight gain in pregnancy (g)**		
1st week	19.75 ± 5.96	20.42 ± 7.95
2nd week	22.75 ± 10.60	20.75 ± 4.90
3rd week	79.67 ± 15.76	79.67 ± 15.76
Total body weight gain—WG	121.1 ± 12.2	115.3 ± 19.0
Gravid uterus weight—GUW (g)	72.5 ± 19.3	79.4 ± 12.1
BWG minus GUW (g)	44.27 ± 14.96	38.34 ± 10.65
Daily food consumption (g)	20.32 ± 2.11	21.39 ± 2.60
Daily water intake (mL)	55.55 ± 6.96	56.48 ± 12.64
**Relative organ weight (g/100g)**		
Heart	0.31 ± 0.03	0.31 ± 0.03
Liver	3.94 ± 0.41	3.98 ± 0.31
Spleen	0.22 ± 0.06	0.20 ± 0.02
Kidneys	0.55 ± 0.04	0.55 ± 0.03
**Biochemical serum parameters**		
ALT (U/L)	41.41 ± 10.61	39.50 ± 8.79
AST (U/L)	145.82 ± 32.42	127.98 ± 42.13
Urea (mg/dL)	45.43 ± 8.69	49.80 ± 7.70
Creatinine (mg/dL)	0.62 ± 0.16	0.58 ± 0.12

Data shown as mean ± standard deviation (SD). *p* > 0.05 compared with the Control group (Student’s *t*-test).

## Data Availability

Not applicable.

## Supplementary Materials

The following supporting information can be downloaded at: https://www.mdpi.com/article/10.3390/polym15092163/s1, Figure S1: The UV-vis spectra precursors and control samples title; Figure S2: Changes in the maximum absorption of the plasmonic band during the reaction time title; Figure S3: The nanocomposites thermogravimetric analysis title; Figure S4: Nanoparticle diameter histogram title; Figure S5: Scattered light intensity distribution profiles as a function of the hydrodynamic diametertitle; Figure S6: Effect of PLU on viability of the NIH-3T3 cells determined by MTT assay title.
