# Supplementary material for: UV-Accelerated Synthesis of Gold Nanoparticle–Pluronic Nanocomposites for X-ray Computed Tomography Contrast Enhancement
Source: Polymers (Basel). 2023 May 1;15(9):2163. doi: 10.3390/polym15092163 (PMC10181159; doi:10.3390/polym15092163)
Supplement: Supplementary file 1 [file polymers-15-02163-s001.zip › polymers-2284812-supplementary.pdf]

2284812 Supplementary Material:

UV-Accelerated Synthesis of Gold nanoparticle-Pluronic Nanocomposites for X-Ray Computed Tomography Contrast Enhancement

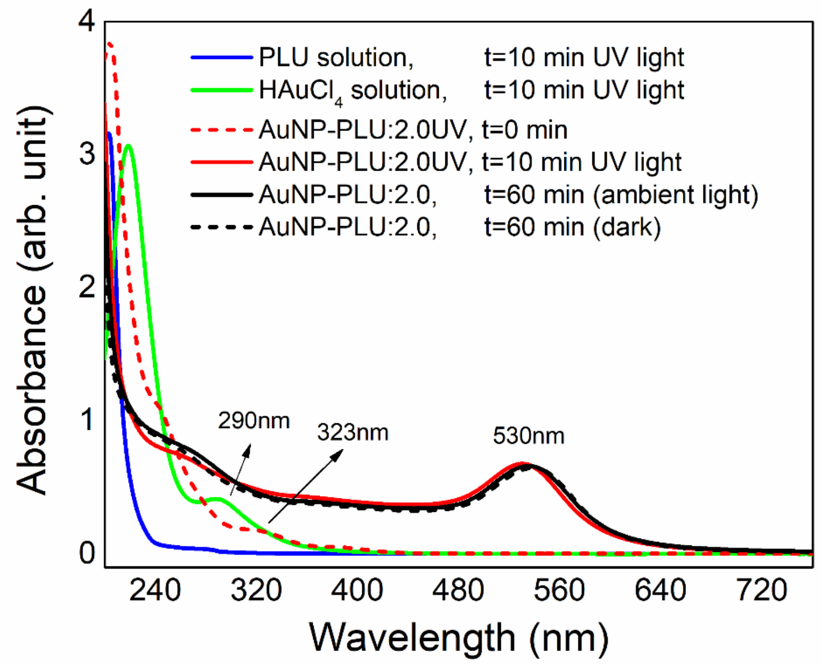

Figure S1. UV-vis spectra obtained solutions of $HAuCl_4$ and PLU at concentrations of 2 mmol L^{-1}; the AuNP-PLU:2.0UV nanocomposite at t=0 (after mixing the precursors) and the formation of the AuNP-PLU nanocomposite is observed after 10 min; and the control samples AuNP-PLU:2.0 (ambient light and in the dark) after 60 min of mixing.

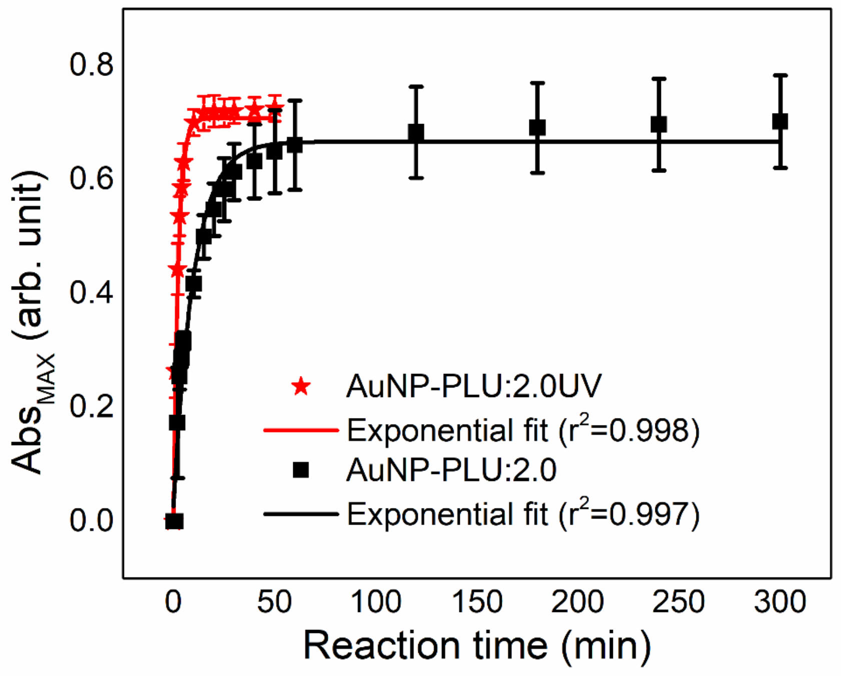

Figure S2. Changes in the maximum absorption AuNP-PLU:2.0UV (red) and AuNP-PLU:2.0 (black) of the plasmonic band during the reaction time. Data of (AuNP-PLU:2.0 were reproduced with permission [32]. Copyright 2018, Elsevier B. V.

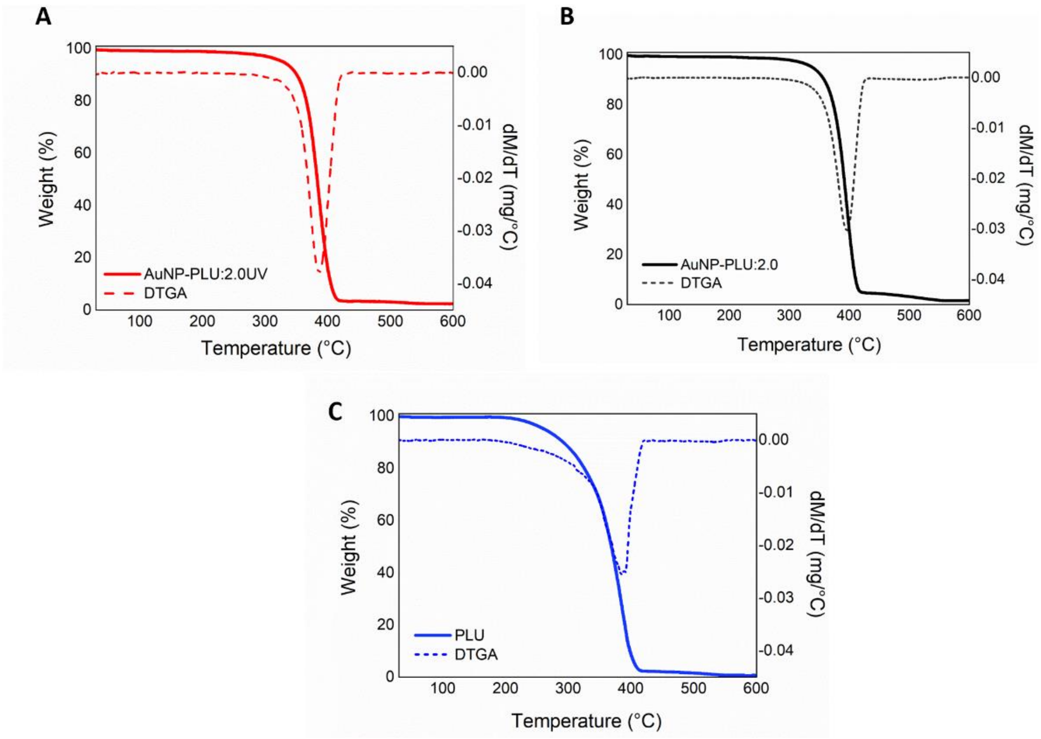

Figure S3. The nanocomposites thermogravimetric analysis. The thermogravimetric curves and the derivative thermogravimetric curves for AuNP-PLU:2.0UV, (A) AuNP-PLU:2.0 (B) and PLU (C)

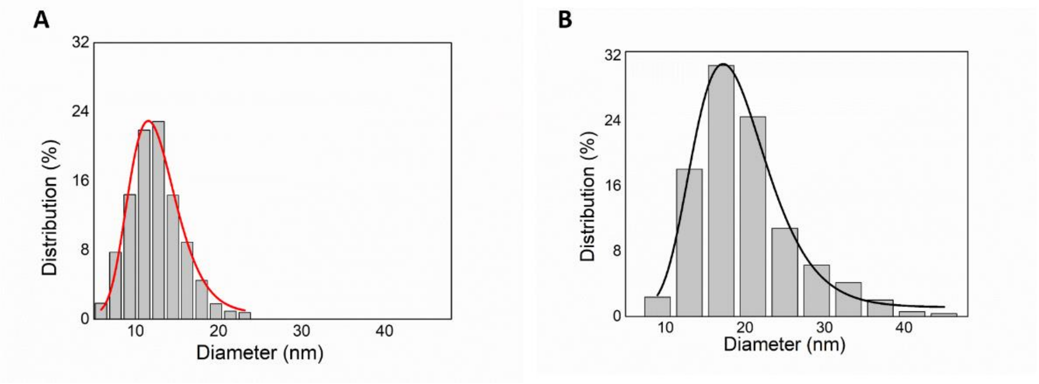

Figure S4. Nanoparticle diameter histogram (vertical bars) obtained from TEM images, fitted with a log-normal distribution function (solid line) for AuNP-PLU:2.0UV in (A) and AuNP-PLU:2.0 in (B).

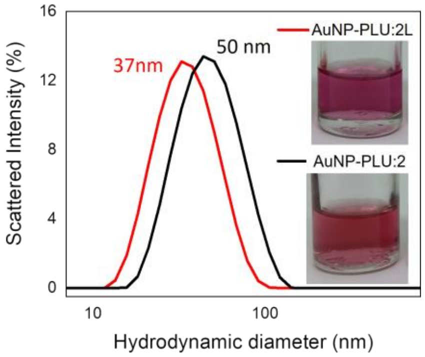

Figure S5. Scattered light intensity distribution profiles as a function of the hydrodynamic diameter of the AuNP-PLU:2.0UV (red line) and AuNP-PLU:2.0 (black line). Inset: digital images of colloidal dispersions, as indicated.

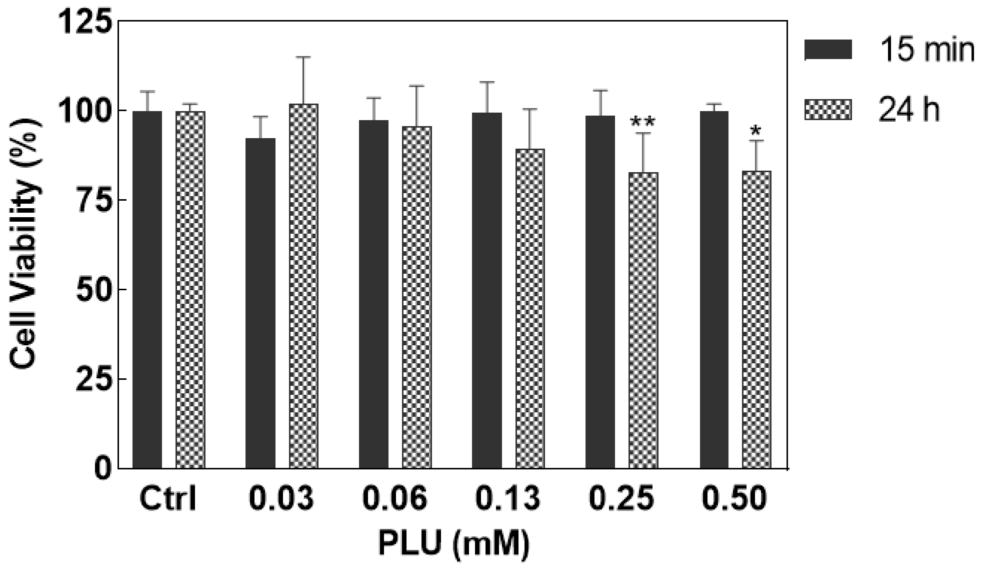

Figure S6. Effect of PLU on viability of the NIH-3T3 cells determined by MTT assay**.** Cells were incubated with PLUfor 15 minutes or 24 hours. Data were presented as mean ± standard deviation (SD) from three independent experiments. Differences among groups were determined by Kruskal-Wallis test and Dunn's multiple comparison post-hoc test. Asterisks indicate significant differences compared to the respective control group: $^{*}p < 0.05$, $^{***}p < 0.001$, $^{****}p < 0.0001$.
